# Dielectric Permittivity, AC Electrical Conductivity and Conduction Mechanism of High Crosslinked-Vinyl Polymers and Their Pd(OAc)_2_ Composites

**DOI:** 10.3390/polym13173005

**Published:** 2021-09-05

**Authors:** Elsayed Elbayoumy, Nasser A. El-Ghamaz, Farid Sh. Mohamed, Mostafa A. Diab, Tamaki Nakano

**Affiliations:** 1 Chemistry Department, Faculty of Science, Damietta University, New Damietta 34517, Egypt; faridsh@mans.edu.eg (F.S.M.); m_adiab@du.edu.eg (M.A.D.); 2 Physics Department, Faculty of Science, Damietta University, New Damietta 34517, Egypt; Elghamaz@du.edu.eg; 3 Institute for Catalysis and Graduate School of Chemical Sciences and Engineering, Hokkaido University, N21 W10, Kita-ku, Sapporo 001-0021, Japan; 4 Integrated Research Consortium on Chemical Sciences (IRCCS), Institute for Catalysis, Hokkaido University, N21 W10, Kita-ku, Sapporo 001-0021, Japan

**Keywords:** metal polymer composites, electrical conductivity, dielectric permittivity, semiconductors, high crosslinked-vinyl polymers

## Abstract

Semiconductor materials based on metal high crosslinked-vinyl polymer composites were prepared through loading of Pd(OAc)_2_ on both Poly(ethylene-1,2-diyl dimethacrylate) (poly(EDMA)) and poly(ethylene-1,2-diyl dimethacrylate-co-methyl methacrylate) (Poly(EDMA-co-MMA)). The thermochemical properties for both poly(EDMA) and poly(EDMA-co-MMA) were investigated by thermal gravimetric analysis TGA technique. The dielectric permittivity, AC electrical conductivity and conduction mechanism for all the prepared polymers and their Pd(OAc)_2_ composites were studied. The results showed that the loading of polymers with Pd(OAc)_2_ led to an increase in the magnitudes of both the dielectric permittivity and AC electrical conductivity (*σ_ac_*). The value of *σ_ac_* increased from 1.38 × 10^−5^ to 5.84 × 10^−5^ S m^−1^ and from 6.40 × 10^−6^ to 2.48 × 10^−5^ S m^−1^ for poly(EDMA) and poly(EDMA-co-MMA), respectively, at 1 MHz and 340 K after loading with Pd(OAc)_2_. Additionally, all the prepared polymers and composites were considered as semiconductors at all the test frequencies and in the temperature range of 300–340 K. Furthermore, it seems that a conduction mechanism for all the samples could be Quantum Mechanical Tunneling (QMT).

## 1. Introduction

Polymers have attracted a lot of interest in the development of modern technologies due to their easy synthesis, cheaper cost, high stability, noncorrosive nature, and low density, which makes them suitable materials for replacing metals and ceramics [1,2]. However, the electrical insulating nature of most polymers, their electrical conductivity σ_ac_ in the range of 10^−12^–10^−16^ S m^−1^, has limited their technological and engineering applications. The insulating nature of the polymers can be improved by introducing highly electrically conductive fillers to the polymer matrices, having σ_ac_ values in the range of 10^2^–10^7^ S m^−1^, which can facilitate the movement of charge carriers through the electron hopping or tunneling process [3]. These functional filler materials include carbon-based nanomaterials, ceramics, metals, or metal oxides [4]. As a result, semiconductors based on polymer composites with electrical properties close to metals and mechanical properties like plastics were produced [5,6,7]. These semiconductor composites can be applied in many electronic applications involving an electrostatic dissipation (ESD) apparatus, electromagnetic interference (EMI) shielding, electrostatic paints, supercapacitors, lightning strike protection, electro-optical devices, the packaging of electronic devices and bipolar plates in the application of fuel cells [8,9,10,11,12,13].

Here are some instances of the most used metals as filler materials to produce semiconductor polymer composites. When a polyethylene matrix was filled with Cu(Cu_2_O) as spherical nanoparticles with a size of 10–25 nm, its electrical conductivity increased by about 4.5–5 times [14]. Nickel was used as a filler material for enhancing the electrical, mechanical and thermal properties of epoxy polymer (EP) [15]. Polydimethylsiloxane (PDMS) was infiltrated into an Au@CNT/sodium alginate sponge skeleton to give Au@CNT/sodium alginate/polydimethylsiloxane flexible composites which had a better electrical conductivity than PDMS itself [16]. Silver was introduced into chitosan/dimethyl amino ethyl methacrylate (chitosan-g-PDMAEMA) with different concentrations, and the results exhibited that the electrical conductivity of the chitosan-g-PDMAEMA/Ag^+^ (2%) composite was better than the original chitosan-g-PDMAEMA [17]. A semiconductor polymer nanocomposite film was based on the doping Poly(vinylidene fluoride) (PVDF) and Poly(vinyl Chloride) (PVC) with different concentrations of palladium nanoparticles (PdNPs) using laser ablation technique, and the results showed that the presence of PdNPs improved the electrical conductivity of PVDF/PVC [18]. PdNPs were used as a filler for few-walled carbon nanotubes (FWCNTs) to produce a transparent and highly electrically conductive film which was applied for the reduction reaction of H_2_. The results exhibited an extremely lower sheet resistance of the poly(ethylene terephthalate) substrates coated with Pd@f-FWCNTs than FWCNTs by about 1/25 [19]. Other metals and oxides including Fe, Al, I_2_, V_2_O_5_ and CdS were used as filler materials for the production of conductive MPCs [20,21,22,23,24].

In our previous work, Pd nanoparticles were loaded on Poly(ethylene-1,2-diyl dimethacrylate) (poly(EDMA)) and poly(ethylene-1,2-diyl dimethacrylate-co-methyl methacrylate) (Poly(EDMA-co-MMA)) and applied as a heterogenous catalyst for the oxidation of benzyl alcohol to benzaldehyde and toluene. The formed Pd-polymer catalysts were characterized by XRD, TEM, and nitrogen gas adsorption [25].

In the present work, we will investigate the influence of Pd loading of both poly(EDMA) and poly(EDMA-co-MMA) on dielectric permittivity and electrical conductivity as well as study the suitable conduction mechanism.

## 2. Materials and Methods

### 2.1. Materials

α,α’-Azobisisobutylonitrile (AIBN), tetrahydrofuran (THF), ethylene-1,2-diyl dimethacrylate (EDMA), and methyl methacrylate (MMA) were purchased from Wako Chemical (Osaka, Japan). Palladium acetate was purchased from Sigma-Aldrich (St. Louis, MO, USA). AIBN was purified by recrystallization from ethanol before use. THF was purified by distillation before use. All the other chemicals and organic solvent (general grade) were received from commercial sources and used directly as received without any further purification.

### 2.2. Synthesis of poly(EDMA) and poly(EDMA-co-MMA)

**Scheme 1:**
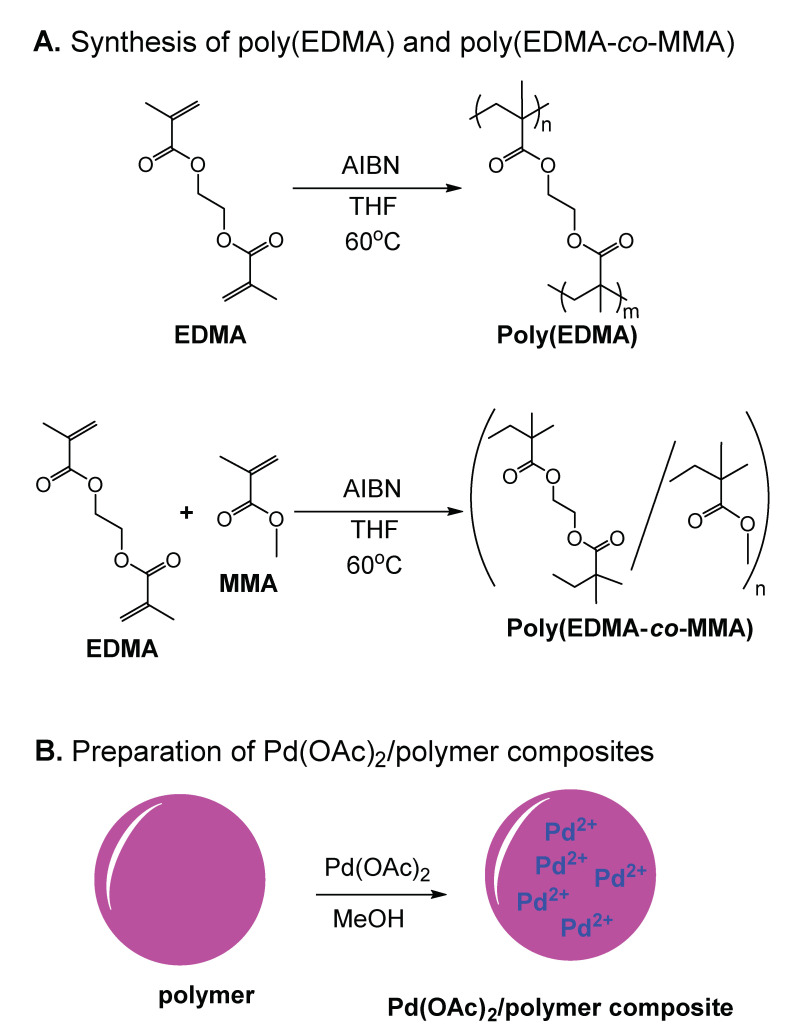
(**A**) Synthesis of poly(EDMA) and poly(EDMA-co-MMA) and (**B**) preparation of Pd(OAc)_2_/polymer composites.

Poly(ethylene-1,2-diyl dimethacrylate) (poly(EDMA)) and poly(ethylene-1,2-diyl dimethacrylate-co-methyl methacrylate) (Poly(EDMA-co-MMA)) were prepared according to the procedure described in our previous work [25] by free radical polymerization of EDMA monomer and copolymerization of EDMA and MMA monomers using AIBN as the initiator in the presence of THF as the solvent (Scheme 1A). To prepare poly(EDMA), a two-necked round bottom flask (200 mL) connected with a condenser was charged with AIBN (0.082 gm, 0.5 mmol), evacuated, and filled with nitrogen gas three times. THF (100 mL), EDMA (4.94 gm, 25 mmol) were then added to the flask with stirring, and a homogenous solution was produced. The reaction mixture was heated at 60 °C under nitrogen atmosphere for 24 h. The reaction was quenched by cooling the reaction solution to room temperature. The produced poly(EDMA), which was insoluble in the solution, was collected by centrifuge and washed with methanol and acetone several times to remove the unpolymerized monomer and initiator. Finally, the purified polymer was dried under vacuum for 24 h. The yield was 4.85 gm (>99%) as a white solid crystal. For poly(EDMA-co-MMA), the same method was used, and the amounts of EDMA and MMA were (5.30 gm, 26 mmol) and (2.67 gm, 26 mmol), respectively. The yield of poly(EDMA-co-MMA) was 7.95 gm (>99%) as a white solid crystal.

### 2.3. Loading of the Polymeric Materials with Pd(OAc)_2_

Pd(OAc)_2_ was loaded on poly(EDMA) and poly(EDMA-co-MMA) by soaking polymeric materials in a solution of Pd(OAc)_2_ in methanol according to the procedure described in our previous work [25] (Scheme 1B). Each polymer, poly(EDMA) and poly(EDMA-co-MMA), (150 mg) was added to a solution of Pd(OAc)_2_ (10 mg, 0.044 mmol) in methanol (50 mL) and stirred for 12 h to allow Pd(OAc)_2_ molecules to go deep within the polymer matrices. After soaking for 12 h, Pd(OAc)_2_/poly(EDMA) and Pd(OAc)_2_/poly(EDMA-co-MMA) were separated from the unloaded Pd(OAc)_2_ solution by centrifuge and washed with methanol several times to ensure that the unloaded Pd(OAc)_2_ was removed from the polymers. Finally, Pd(OAc)_2_/poly(EDMA) and Pd(OAc)_2_/poly(EDMA-co-MMA) were dried under vacuum for 24 h, and the weight of the two composites became constant, which indicated the complete removal of all solvent molecules (the yield was 157.7 mg and 152.4 mg as black solid crystals for Pd(OAc)_2_/poly(EDMA) and Pd(OAc)_2_/poly(EDMA-co-MMA), respectively). The loaded amount of Pd(OAc)_2_ which was immobilized on poly(EDMA) and poly(EDMA-co-MMA) was determined by gravimetry after washing and drying the composites and was found to be 5.1% and 1.6% of the weight of poly(EDMA) and poly(EDMA-co-MMA), respectively. The structure and chemical properties of poly(EDMA), poly(EDMA-co-MMA) and their Pd composites were discussed in detail in our previous paper [25].

### 2.4. Measurements

Thermal gravimetric analysis (TGA) was carried out by using Rigaku Thermo plus TG8120 and DSC8230 apparatuses under the flow of nitrogen gas (20 mL/min) and a heating rate of 10 K/min on an aluminum crucible from room temperature to 750 K. The AC electrical conductivity was measured by Hioki 3532-50 LCR hitester in a temperature range of 300–400 K. The samples were comprised of a pellet with a thickness of 1 mm and a surface area of 1.3 cm^2^. During the measurements, the samples were fixed between two copper electrodes.

## 3. Results and Discussion

### 3.1. Thermal Gravimetric Analysis

**Figure 1:**
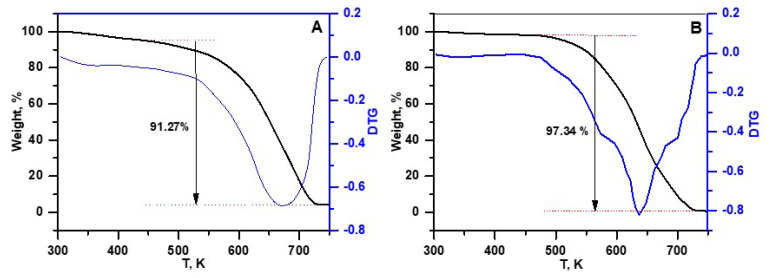
TGA and DTG of (**A**) poly(EDMA) and (**B**) poly(EDMA-co-MMA).

The thermal gravimetric analysis (TGA) and derivative thermal gravimetric (DTG) of poly(EDMA) and poly(EDMA-co-MMA) are presented in Figure 1. The TGA curves for both polymers showed a slight decrease in weight loss percentage starting from 340 K to about 500 K; then, the weight loss increased by higher rates from about 500 K to 730 K. The DTG curves for both polymers show a main degradation peak at temperatures of 670 K and 640 K with weight loss percentages of 91.27% and 97.34% for poly(EDMA) and poly(EDMA-co-MMA), respectively. The low degradation weight loss percentage starting at 340 K in both polymers could be attributed to a loss of moisture or residual organic solvents from the matrices, while the high-rate degradation weight loss percentage starting at about 500 K could be attributed to the degradation of the backbone of both polymers. In addition, the two polymers are chemically stable up to 460 K and 480 K for poly poly(EDMA) and poly(EDMA-co-MMA), respectively.

The activation energy and thermodynamic parameters of the main thermal stage degradation for poly(EDMA) and poly(EDMA-co-MMA) are determined by using the Coats–Redfern method [26,27]. According to Coats–Redfern, the mathematical formula of the first-order reaction is given by Equation (1):

(1) $$\log\left[\frac{-\log\left(1-\alpha \right)}{T^{2}}\right]=\log\left[\frac{A^{\prime }R}{\theta E^{*}}\left(1-\frac{2RT}{E^{*}}\right)\right]-\frac{E^{*}}{2.303RT},$$

where *α* is the fraction of sample decomposed at temperature *T*, *A*′ is the Arrhenius constant, *R* is the general gas constant, *θ* is the heating rate and E^*^ is the activation energy. The value of *α* is calculated according to Equation (2):

(2) $$\alpha =\frac{W_{o}-W_{t}}{W_{o}-W_{f}}\;,$$

where *W_o_*, *W_t_* and *W_f_* are the initial weight of the sample, weight of the sample at any given temperature and the final weight of the sample after completion of the reaction, respectively. By applying Equation (1) on the experimental data of TGA and plotting the relation between $\log\left[\frac{-\log\left(1-\alpha \right)}{T^{2}}\right]$ and 1/*T*, a straight line was produced for both poly(EDMA) and poly(EDMA-co-MMA), and the values of E^*^ and *A*^′^ were calculated from the slope and intercept with the Y-axis (Figure S1 in the supporting information).

The entropy (∆S^*^), enthalpy (∆H^*^), and change in free energy (∆G^*^) of the activation are determined for both poly(EDMA) and poly(EDMA-co-MMA) by Equations (3)–(5) [28]:

(3) $$\Delta S^{*}=2.303R\left[\log\left(\frac{A^{\prime }h}{K_{B}T}\right)\right],$$

(4) $$\Delta H^{*}=E^{*}-RT,$$

(5) $$\Delta G^{*}=\Delta H^{*}-T\Delta S^{*},$$

where *h*, *K_B_* are the Planck and Boltzmann constants, respectively. The thermal activation energy, Arrhenius constant and thermodynamic parameters for both poly(EDMA) and poly(EDMA-co-MMA) are summarized in Table 1. From these results, the degradation of both polymers is a nonspontaneous and endothermic process, which is confirmed by the positive values of ∆G^*^ and ∆H^*^, respectively [28].

### 3.2. AC Electrical Properties

#### 3.2.1. Dielectric Permittivity

The dielectric permittivity of a material (*ε*) is considered a complex quantity with a real part (*ε_r_*) and imaginary part (*ε_i_*) and is given by Equation (6) [29,30]:

(6) $$\varepsilon =\varepsilon_{r}+i\varepsilon_{i},$$

The values of the real and imaginary parts of dielectric permittivity *ε_r_* and *ε_i_* are calculated from the value of capacitance measured in parallel mode (*C_p_*) and the loss tangent (tan *δ*). The values of *C_p_* and tan *δ* are measured for poly(EDMA), poly(EDMA-co-MMA) and their Pd(OAc)_2_ composites in the temperature range of 300–400 K and frequency range of 0.5–1000 KHz. The values of *ε_r_* and *ε_i_* are calculated according to the following Equations (7) and (8) [29,30]:

(7) $$\varepsilon_{r}=\frac{C_{p}\;d}{\varepsilon_{o}\;A},$$

(8) $$\varepsilon_{i}=\varepsilon_{r}\tan\left(\delta \right),$$

where *ε_o_* is the permittivity of free space, and *d* and *A* are the sample’s thickness and cross-section area, respectively.

Polymers and other dielectric compounds exhibit several relaxation and loss (dissipation) modes that appear as maxima in the dielectric spectrum depending on the type of material, temperature and the applied electric field. In polymers, relaxation as well as dielectric loss may be due to the motion of relatively long chain movements in the amorphous region. When the frequency of the applied external field is comparable to the rate of the internal motions, the loss function (tan *δ*) and consequently the imaginary dielectric constant (*ε_i_*) tend to increase, and a maximum may be observed [31]. 

**Figure 2:**
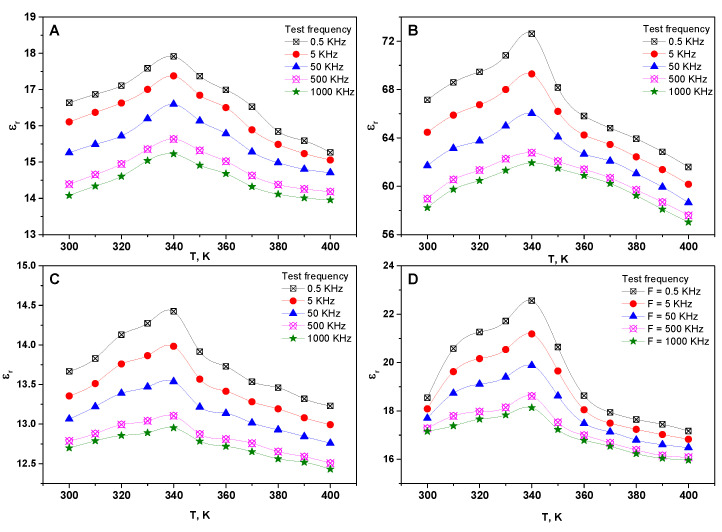
Temperature and frequency dependence on real dielectric constant for (**A**) poly(EDMA), (**B**) Pd(OAc)_2_/poly(EDMA)-5.1%, (**C**) poly(EDMA-co-MMA), and (**D**) Pd(OAc)_2_/poly(EDMA-co-MMA)-1.6%.

**Figure 3:**
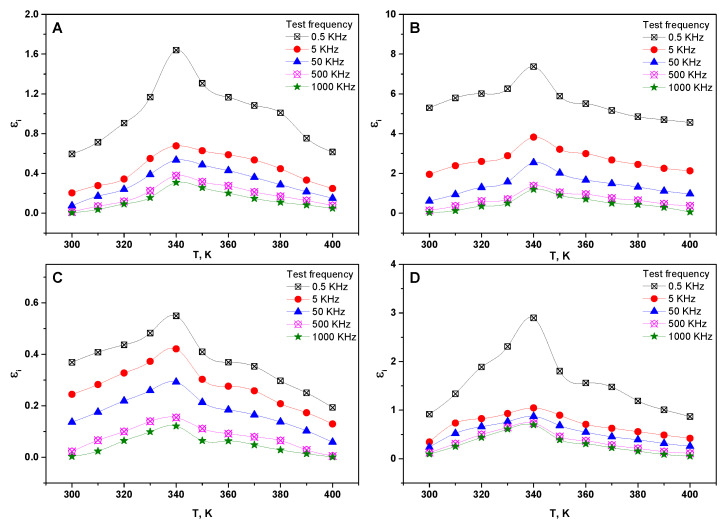
Temperature and frequency dependence on imaginary dielectric constant for (**A**) poly(EDMA), (**B**) Pd(OAc)_2_/poly(EDMA)-5.1%, (**C**) poly(EDMA-co-MMA), (**D**) and Pd(OAc)_2_/poly(EDMA-co-MMA)-1.6%.

The dependences of *ε_r_* and *ε_i_* on temperature and frequency for poly(EDMA), poly(EDMA-co-MMA) and their Pd(OAc)_2_ composites are presented in Figure 2 and Figure 3, respectively. Both *ε_r_* and *ε_i_* decrease when increasing the test frequency from 0.5 to 1000 KHz. On the other hand, the dependences of *ε_r_* and *ε_i_* on temperature exhibit an irregular behavior in the temperature range under consideration for the prepared polymers and their Pd(OAc)_2_ composites (a peak value around 340 K). Taking into account the absence of any peaks in DTG with a slight decrease in TGA curves in the temperature range of 300–500 K, the peak value at this temperature could be attributed to a loss of moisture or residual organic solvents [28,32,33]. Furthermore, the loading of both polymers by Pd(OAc)_2_ leads to an increase in the value of *ε_r_* (Figure 2B,D) and *ε_i_* (Figure 3B,D). This behavior of the dielectric permittivity with the frequency and temperature for both polymers and their Pd(OAc)_2_ composites has been reported for other polymers and organic composites [24,33,34,35,36,37,38,39].

#### 3.2.2. AC Electrical Conductivity

The AC electrical conductivity (*σ_ac_*) is expressed as a function of both the value of the imaginary dielectric permittivity (*ε_i_*) and angular frequency (*ω* = 2πF), and is calculated according to Equation (9) [29,30]:

(9) $$\sigma_{ac}=\omega \varepsilon_{o}\varepsilon_{i},$$

**Figure 4:**
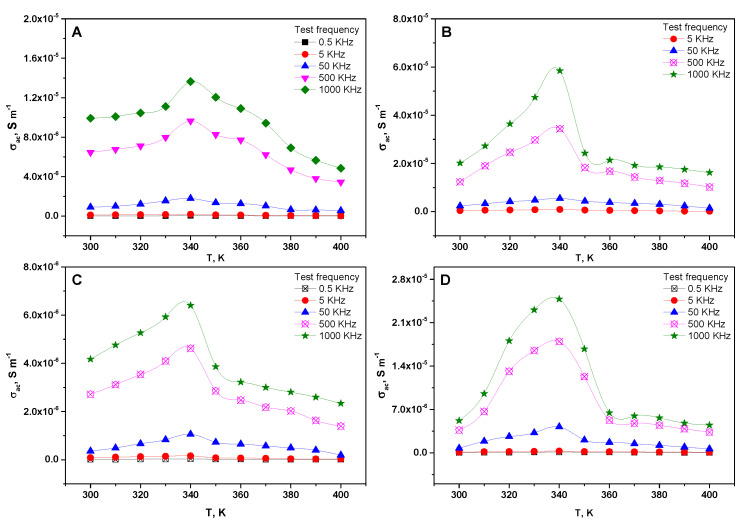
Temperature and frequency dependence on the AC conductivity for (**A**) poly(EDMA), (**B**) Pd(OAc)_2_/poly(EDMA)-5.1%, (**C**) poly(EDMA-co-MMA), and (**D**) Pd(OAc)_2_/poly(EDMA-co-MMA)-1.6%.

Figure 4 shows the dependence of *σ_ac_* on both the temperature in the temperature range of 300–400 K and the test frequency in the range of 0.5 KHz–1 MHz for the prepared polymers and their Pd(OAc)_2_ composites. For all samples under testing, *σ_ac_* increases when increasing the test frequency; however, it demonstrates two different behaviors depending on the temperature. In the first stage of heating up to 340 K, a semiconductor behavior is observed, while at a higher temperature, *σ_ac_* decreases as the temperature increases more (metallic behavior), and this behavior is in agreement with other reported polymers and organic materials [24,32,34,39,40]. On the other hand, the existence of Pd(OAc)_2_ slightly enhances the conductivity of the two polymers. For example, the peak value at a test frequency of 1 MHz for poly(EDMA) was 1.38 × 10^−^^5^ S m^−1^ and increased to 5.84 × 10^−5^ S m^−^^1^ for Pd(OAc)_2_/poly(EDMA)-5.1%. Additionally, it increased from 6.40 × 10^−^^6^ to 2.48 × 10^−5^ S m^−^^1^ for poly(EDMA-co-MMA) and Pd(OAc)_2_/poly(EDMA-co-MMA)-1.6%, respectively. From these results, we can conclude that poly(EDMA), Pd(OAc)_2_/poly(EDMA)-5.1%, poly(EDMA-co-MMA), and Pd(OAc)_2_/poly(EDMA-co-MMA)-1.6% exhibit a semiconductor behavior in the temperature range of 300–340 K at all the test frequencies, with an enhancement of the electrical conductivity value by adding Pd(OAc)_2_ to the polymer matrices.

**Figure 5:**
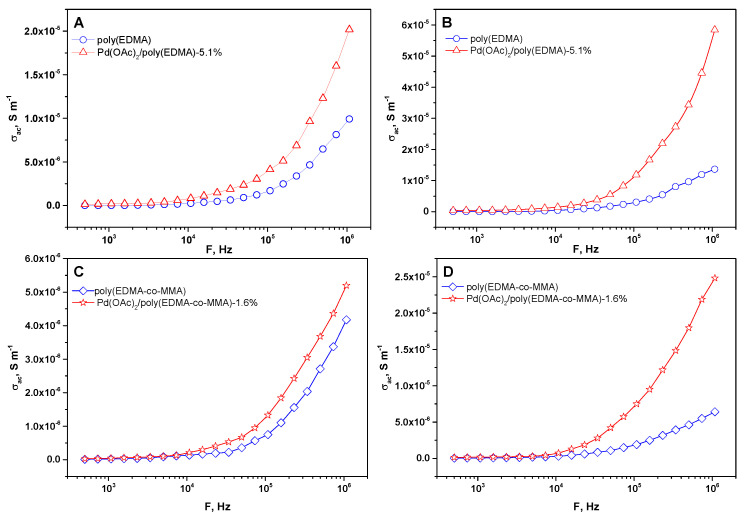
The effect of loading poly(EDMA) and poly(EDMA-co-MMA) with Pd(OAc)_2_ on the AC conductivity at (**A**,**C**) 300 K and (**B**,**D**) 340 K.

Figure 5 illustrates the effect of loading poly(EDMA) and poly(EDMA-co-MMA) with Pd(OAc)_2_ on the electrical conductivities across the entire test frequency range at room temperature (Figure 5A,C) and at 340 K (Figure 5B,D). It appears that the loading of Pd(OAc)_2_ has a stronger effect on *σ_ac_* at a higher temperature and frequency. This result can be explained due to the increase in charge carriers, since the loading of metal ions leads to an increase of free charge carriers, which, accordingly, leads to an increase of electrical conductivity. This result is in good agreement with the results of other polymers doped with different metal ions [18,19,24,33,38,39].

The thermal activation energy (∆*E_ac_*) for the AC electrical conductivity is determined by applying the following Arrhenius equation [28,34]:

(10) $$\sigma_{ac}=\sigma_{o}e^{\left(\frac{-\Delta E_{ac}}{k_{B}T}\right)},$$

where *σ_o_* is the pre-exponential constant and *k_B_* is the Boltzmann constant.

**Figure 6:**
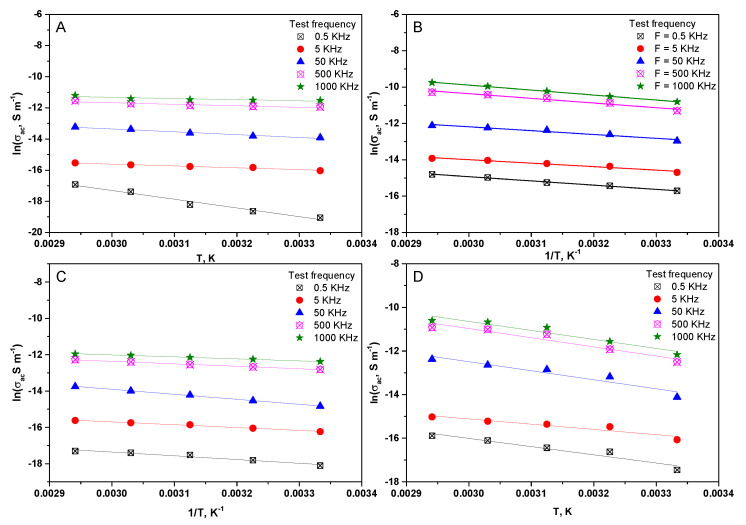
Dependence of ln(σac) on the temperature at different frequencies for (**A**) poly(EDMA), (**B**) Pd(OAc)_2_/poly(EDMA)-5.1%, (**C**) poly(EDMA-co-MMA), and (**D**) Pd(OAc)_2_/poly(EDMA-co-MMA)-1.6%.

**Figure 7:**
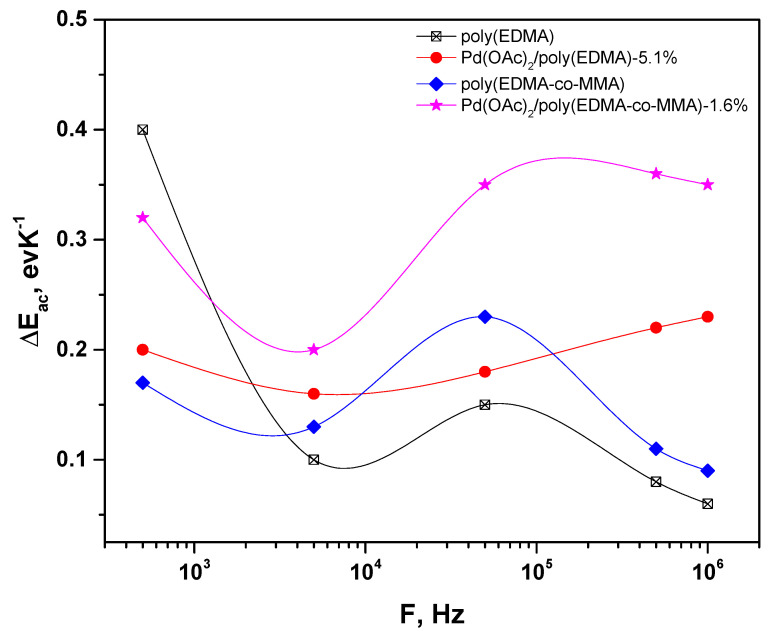
Dependence of ∆Eac on the frequency.

The Arrhenius equation is applied for the experimental data in the temperature range of 300–340 K (semiconductor behavior) and at selected test frequencies for all specimens under consideration. Figure 6 shows the relation between ln(*σ_ac_*) and 1/T for all the prepared polymers and their Pd(OAc)_2_ composites. As seen from Figure 6, straight lines with negative slopes are produced, in which ∆*E_ac_* can be calculated according to Equation (10). Figure 7 presents the dependence of ∆*E_ac_* on the frequency for poly(EDMA), Pd(OAc)_2_/poly(EDMA)-5.1%, poly(EDMA-co-MMA) and Pd(OAc)_2_/poly(EDMA-co-MMA)-1.6%. It is clear from this figure that ∆*E_ac_* has values in the range of 0.1–0.4 eV, depending on both the test frequency and the type of polymeric material or composite.

#### 3.2.3. Conduction Mechanism

The conduction mechanism of semiconductors is investigated through theoretical models based on the dependence of the AC electrical conductivity on the test frequency according to the following Equation (11):

(11) $$\sigma_{ac}=A\omega^{s},$$

where *A* is a constant depending on the temperature, and *S* is the exponent whose value and behavior with the temperature determine the suitable conduction mechanism. The most frequently reported conduction mechanisms are the small polaron tunneling (SPT) model, correlated barrier hopping (CBH) model and quantum mechanical tunneling (QMT) model [29,30]. In the SPT model, the value of *S* and *σ_ac_* are calculated according to the following Equations (12) and (13):

(12) $$S_{\left(SPT\right)}=1-\frac{4}{\ln\left[\frac{1}{\omega \tau_{o}}\right]-\frac{w_{H}}{k_{B}T}},$$

(13) $$\sigma_{ac\left(SPT\right)}=\frac{\pi^{4}}{24}\frac{e^{2}k_{B}T\left[N(E_{f}\right){]}^{2}\omega R_{w}^{\prime 4}}{\alpha },$$

where *W_H_* is the barrier height for infinite site separation, *τ_o_* is the relaxation time, *e* is the charge of electron, *a* is the spatial extent of polaron, *N(E_F_)* is the density of states at the Fermi level and *R^′^_w_* is the tunneling distance. For the CBH model, the electrons in charged defect states hop over the Coulomb barrier with height *W,* which is calculated according to Equation (14):

(14) $$W_{\left(CBH\right)}=W_{m}-\frac{e^{2}}{{\pi \varepsilon \varepsilon }_{o}R},$$

where *W_m_* and *R* are the maximum barrier height and the distance between hopping states, respectively. The values of *S*, *σ_ac_* and the hopping length (*R_ω_*) are determined using Equations (15)–(17):

(15) $$\sigma_{ac\left(CBH\right)}=\frac{1}{24}\pi^{3}N^{2}\varepsilon \varepsilon_{o}\omega R_{\omega }^{6},$$

(16) $$R_{\omega }{}_{\left(CBH\right)}=\frac{ne^{2}}{\pi \varepsilon \varepsilon_{o}}[W_{m}-k_{B}Tln\left(\frac{1}{\omega \tau_{o}}\right)],$$

(17) $$S_{\left(CBH\right)}=1-\frac{6k_{B}T}{W_{m}-k_{B}Tln\left(\frac{1}{\omega \tau_{o}}\right)},$$

According to the QMT model, *S* is independent of temperature and is given by Equation (18):

(18) $$S_{\left(QMT\right)}=1-\frac{4}{\ln\left[\frac{1}{\omega \tau_{o}}\right]},$$

**Figure 8:**
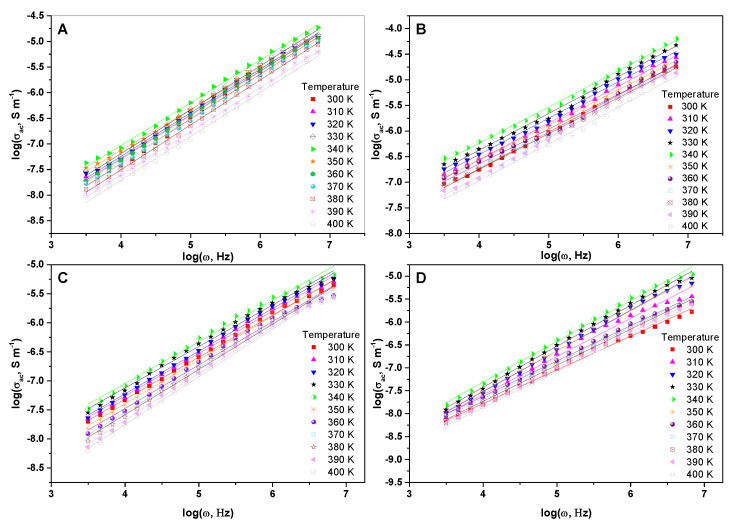
Dependence of log(σ_ac_) on the frequency at different temperatures for (**A**) poly(EDMA), (**B**) Pd(OAc)_2_/poly(EDMA)-5.1%, (**C**) poly(EDMA-co-MMA), and (**D**) Pd(OAc)_2_/poly(EDMA-co-MMA)-1.6%.

**Figure 9:**
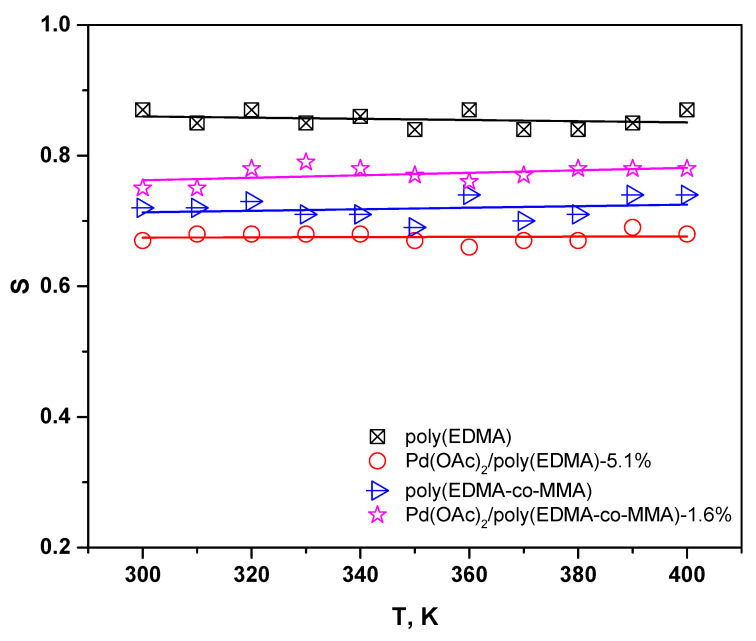
Dependence of the S value on the temperature.

Figure 8 shows the relation between log(*σ_ac_*) and log(*ω*) at the temperature range of 300–400 K. This relationship produces a straight line in which its slope is equal to the value of *S*. The behavior of *S* with the temperature suggests the probable conduction mechanism for the materials. The dependence of *S* on the temperature for all the prepared polymers and their Pd(OAc)_2_ composites is presented in Figure 9. It is clear that *S* is nearly independent of the temperature for all polymers and composites and has values in the range of 0.67–0.85 depending on the polymer type. This behavior and these values of *S* strongly point to Quantum Mechanical Tunneling (QMT) being the best conduction mechanism (Equation (18)) to describe the electrical conduction process in all the samples under consideration [29,30]. QMT has been reported to be the operating conduction mechanism in many polymers and organic compounds such as copolymer (N, N′-bissulphinyl-m-benzenediamine-p-phenylenediamine) [28], quinoline Schiff base complexes [35] and 2-Hydroxy-1-naphthylideneaniline [40].

## 4. Conclusions

In conclusion, poly(EDMA), poly(EDMA-co-MMA) and their Pd(OAc)_2_ were confirmed as semiconductor materials based on the dependence of their *σ_ac_* on both the temperature and test frequency. The TGA analysis showed that the polymers were chemically stable up to 460 K and 480 K for poly(EDMA) and poly(EDMA-co-MMA), respectively. Poly(EDMA), Pd(OAc)_2_/poly(EDMA)-5.1%, poly(EDMA-co-MMA), and Pd(OAc)_2_/poly(EDMA-co-MMA)-1.6% exhibited a semiconductor behavior in the temperature range of 300–340 K at all the tested frequencies, with an enhancement of the value of the electrical conductivity by adding Pd(OAc)_2_ to the polymer matrices. The value of *σ_ac_* increased from 1.38 × 10^−5^ to 5.84 × 10^−5^ S m^−1^ and from 6.40 × 10^−6^ to 2.48 × 10^−5^ S m^−1^ for poly(EDMA) and poly(EDMA-co-MMA), respectively, at 1 MHz and 340 K after loading Pd(OAc)_2_. The activation energy of the electrical conductivity ∆*E_ac_* for all the polymers and their Pd(OAc)_2_ composites showed values in the range of 0.1–0.4 eV, depending on both the test frequency and the type of polymeric material or composite. The dependence of the S values on the temperature strongly points to Quantum Mechanical Tunneling (QMT) being the best conduction mechanism to describe the electrical conduction process in all the samples under consideration.

## Figures and Tables

**Table 1:** Thermal activation energy and thermodynamic parameters of poly(EDMA) and poly(EDMA-co-MMA).

Polymer	E* ^a^ (KJ mol^−1^)	*A*^′^^a^ (S^−1^)	∆S* ^b^ J mol^−1^ K^−1^	∆H* ^b^ (KJ mol^−1^)	∆G* ^b^ (KJ mol^−1^)
Poly(EDMA)	58.55	66.13	−214.88	54.14	168.02
Poly(EDMA-co-MMA)	54.81	38.69	−218.34	50.90	153.52

^a^ calculated from the slope and intercept of the relationship between $\log\left[\frac{-\log\left(1-\alpha \right)}{T^{2}}\right]$ and 1/T (Figure S1 in the supporting information). ^b^ calculated according to Equations (3)–(5).

## Data Availability

Data available in a publicly accessible repository.

## Supplementary Materials

The following are available online at https://www.mdpi.com/article/10.3390/polym13173005/s1, Figure S1: Coats-Redfern relationship for (**A**) poly(EDMA) and (**B**) poly(EDMA-co-MMA).
