# Supplementary material for: Dielectric Permittivity, AC Electrical Conductivity and Conduction Mechanism of High Crosslinked-Vinyl Polymers and Their Pd(OAc)2 Composites
Source: Polymers (Basel). 2021 Sep 5;13(17):3005. doi: 10.3390/polym13173005 (PMC8434503; doi:10.3390/polym13173005)
Supplement: Supplementary file 1 [file polymers-13-03005-s001.zip › polymers-1355195-supplementary.pdf]

Supporting information

Dielectric permittivity, AC electrical conductivity and conduction mechanism of high crosslinked-vinyl polymers and their $Pd(OAc)_2$ composites

Elsayed Elbayoumy [1,*], Nasser A. El-Ghamaz [2], Farid Sh. Mohamed [1], Mostafa A. Diab [1], and Tamaki Nakano [3,4,*]

[1] Chemistry department, Faculty of Science, Damietta University, New Damietta 34517, Egypt;.

[2] Physics department, Faculty of Science, Damietta University, New Damietta 34517, Egypt;.

[3] Institute for Catalysis and Graduate School of Chemical Sciences and Engineering, Hokkaido University, N21 W10, Kita-ku, Sapporo 001-0021, Japan;

[4] Integrated Research Consortium on Chemical Sciences (IRCCS), Institute for Catalysis, Hokkaido Uni-versity, N21 W10, Kita-ku, Sapporo 001-0021, Japan

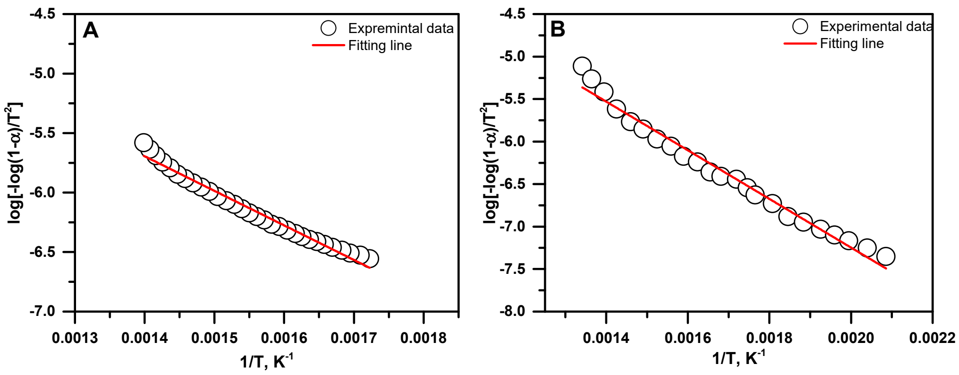

Figure S1. Coats-Redfern relationship for poly(EDMA) (**A**) and poly(EDMA-co-MMA) (**B**).
